# Senescent Cells in IPF: Locked in Repair?

**DOI:** 10.3389/fmed.2020.606330

**Published:** 2020-12-18

**Authors:** Silke Meiners, Mareike Lehmann

**Affiliations:** ^1^Helmholtz Zentrum München, Comprehensive Pneumology Center (CPC), Member of the German Center for Lung Research (DZL), University Hospital, Ludwig-Maximilians University, Munich, Germany; ^2^Research Unit Lung Repair and Regeneration, Helmholtz Zentrum München, Member of the German Center for Lung Research (DZL), Munich, Germany

**Keywords:** senescence, IPF, IPF—idiopathic pulmonary fibrosis, repair, regeneration

## Introduction

Cellular senescence has been recognized since the 1960s as a cell biological program of aging. It also serves physiological functions in organismal development, regeneration and tissue repair. Senescence is triggered by replicative telomere attrition but also stress such as DNA damage, hypoxia, nutrient deprivation, mitochondrial impairment and oncogene activation (1, 2). In response to macromolecular damage, senescent cells enter a presumably permanent cell cycle arrest characterized by activation of the p53 DNA-damage response pathway and transcriptional induction of the cell cycle inhibitors p21WAF1/Cip and p16INK4A, which becomes reinforced by heterochromatin changes (3). Senescent cells are metabolically active, produce senescent-associated β-galactosidase and acquire a senescent-associated secretory phenotype (SASP) with secretion of pro-inflammatory cytokines, proteases and growth factors (3, 4).

The diverse biological functions of senescence in aging, developmental processes, tissue repair, cancer growth and chronic diseases suggest that the senescent state reflects a dynamic cellular stress program depending on the cell type, nature of inducer and extent of senescence with highly variable SASP composition (2, 3, 5). Data from chemo-resistant tumors demonstrated re-entry of senescent cells and their reprogramming into cancer stem cells (6, 7). Similarly, novel single cell RNA sequencing (scRNAseq) analyses of lung repair identified activation of the senescence program in stem cell-like repair cells in mice (8, 9). These data thus challenge our traditional understanding of senescence in repair and disease. They also question whether the term “senescence” is still appropriate or whether we should rather specify the distinct physiological states associated with senescence of different cell types.

## Senescence In Lung Health and Disease

### Senescence in Repair and Development

The timely and spatially controlled induction of senescence is part of a developmental program where the secretory function of senescent cells serves to fine-tune cell fate determination and tissue patterning (10). In highly regenerative organisms, e.g., salamanders and zebrafish, senescent cells contribute to the regeneration of complex structures (11). A recent report suggests the presence of senescent fibroblasts in the developing and adult lung supporting epithelial progenitor cell function (12).

In tissue remodeling and repair, senescence is induced locally in a well-controlled manner (10, 11). The inducing factors are not fully defined. While activation of the senescence program restricts cellular reprogramming in inducible pluripotent stem cells (iPSCs) (13), the senescent cells activate proliferation and reprogramming in neighboring cells via secretion of SASP factors (10, 13, 14). The local induction of cellular stemness by senescent cells contributes to tissue repair and wound healing. It also represents a trade-off of senescence in cancer and might further promote cancer growth and resistance toward chemotherapy (2, 15). In this mode of reparative senescence, the SASP producing senescent cells are cleared by the immune system such as natural killer (NK) cells or macrophages (2, 10, 16, 17) preventing the spread of senescence within the tissue, known as secondary senescence (5).

### Senescence in Aging and Disease

In aging and in chronic diseases, senescent cells accumulate and persist in the tissue (2). Senescence is induced by repeated or chronic exposure to stress over time. Moreover, senescent cells are ineffectively cleared by an aging or dysfunctional immune system in disease (18, 19). Senescence restricts stem cell and progenitor functions in aging and disease (20). It further aggravates inflammation and tissue fibrosis via secretion of pro-inflammatory and pro-fibrotic SASP (4, 21). The senescence program is further spread though-out the tissue upon activation of secondary senescence (5). As SASP factors can be secreted via extracellular vesicles, this might allow senescent cells to signal not only locally but also systemically (22, 23). Age-related or stress-induced senescence of immune cells impairs clearing of senescent cells and further aggravates accumulation of senescent cells thereby contributing to the vicious cycle that promotes aging and chronic diseases (19). In contrast, enhanced clearance of senescent cells delays age-related disorders in mice (24). All of the above mentioned features of senescence have also been demonstrated in lung aging and age-related lung diseases (25–27). While chronic lung diseases as for example IPF and COPD present pathologically very different diseases, they share aging-associated hallmarks such as changes in ECM, aberrant repair processes and cellular senescence (28). Defining the specific phenotypes of these hallmarks might help to shed light on why they give rise to different pathological diseases.

### Senescence in Lung Repair and Fibrosis

Senescence is a well-recognized feature of lung fibrosis. Patients with mutations in telomere genes, which causes replicative senescence, develop familial forms of idiopathic pulmonary fibrosis (IPF) indicating that senescence causally contributes to the development of lung fibrosis in this subgroup of familial IPF (29). Cellular senescence was also observed in non-familial IPF, namely in lung myofibroblasts, in hyperplastic bronchial epithelial cells and in alveolar epithelial cells (27, 30–35). While different cell types display senescent features in IPF and might exert cell-specific effects (27), we here focus our discussion on epithelial cells as the injured epithelium represents an early trigger for disease development (36). Activation of senescence was confirmed in several mouse models of lung injury and fibrosis (27, 34, 37). Importantly, clearing of senescent cells in mice protected from lung fibrosis (33, 34). Available evidence thus supports the above outlined concept that while senescence is a crucial feature of physiological lung repair, it promotes lung fibrosis upon dysregulation and accumulation of senescent cells in the aging lung. These detrimental effects have been mainly attributed to the paracrine pro-fibrotic effects of the SASP and an impaired clearing of accumulating senescent cells in the lung, as also suggested for other tissues (2, 27, 38).

## Re-Thinking Senescence

The recent scRNA seq data from mouse models of lung repair fundamentally challenge the traditional view that senescent cells are irreversibly growth arrested and act mainly in a paracrine fashion to promote proliferation and reprogramming in neighboring cells. Strunz et al. identified a transitional stem cell state involved in alveolar repair of bleomycin-injured mouse lungs (9). These Keratin8+ (Krt8+) alveolar repair cells are characterized by activation of cell senescence and wound healing programs as well as of the p53, MYC and TNFα/NFκB pathways. The cells originate from either AT2 or activated Club cells and transition toward AT1 cells. EdU pulse labeling of lineage-traced cells indicated that these Krt8+ alveolar repair cells were actively proliferating as confirmed by cell cycle regression analysis and Ki67 co-staining. Very similar, Kobayashi et al., identified a transitional stem cell-like cell en route from AT2 to AT1 cells in mouse organoids and models of lung repair (8). These cells were similarly enriched for cellular senescence, TGFβ signaling and p53 activation signatures as also demonstrated by marker expression on protein level. A similar intermediate repair cell type was identified during alveolar regeneration after bleomycin induced lung injury and inflammatory signaling in mice (39). These studies thus demonstrate the dynamic existence of alveolar stem cell-like cells in physiological lung repair, which are de-differentiated and proliferate but at the same time show markers of senescence and activation of SASP. However, the distinct overlap of proliferation and cellular senescence markers within the same cells remains to be carefully demonstrated on the protein level. While the causal contribution of these cells to adaptive lung repair remains to be determined, these findings contradict our conventional understanding of senescence. The cellular senescence program in these alveolar repair cells involves re-entry into the cell cycle, de-differentiation with stem cell-like properties and SASP secretion. This suggests that the senescent cell is capable of doing the repair job itself and does not (only) act as the sentinel to alert neighboring cells by paracrine SASP signaling. Most probably, senescence is induced as part of the tissue repair program to facilitate cellular de-differentiation and stemness involving autocrine SASP signaling. Importantly, as these cells transition into AT1 cells, there is no need for clearing them by immune cells. These findings extend our current view on senescent cells in repair and regeneration (2, 10). They are fully in line with the observed reprogramming of tumor-derived senescent cells into plastic cells which exhibit features of cancer stemness (7). Escape of such senescent tumor cells from their cell cycle arrest transformed them into “super” cancer stem cells with high tumor initiating potential (15).

Remarkably, a very similar signature of de-differentiation, stemness, and senescence was detected in aberrant basaloid cells of irreversibly remodeled IPF lungs (40, 41). These scRNA seq studies dissected the cellular composition of fibrotic IPF lungs in unprecedented detail. Their RNA and protein data confirmed that the previously described aberrant bronchial cell-derived cells show prominent expression of senescence markers (30, 40, 41). In addition to activation of the senescence program, these newly termed basaloid cells showed enriched gene expression for wound healing programs, activation of p53 and integrin signaling pathways, together with the induction of SOX9-controlled genes, which strongly suggests activation of a distal airway development and repair program (42). It remains to be established whether these IPF-specific basaloid cells are permanently cell cycle arrested and have fully lost their proliferative potential. Importantly, the presence of these cells in irreversibly damaged and remodeled IPF lungs strongly indicates that they have lost their repair capacity.

**Figure 1:**
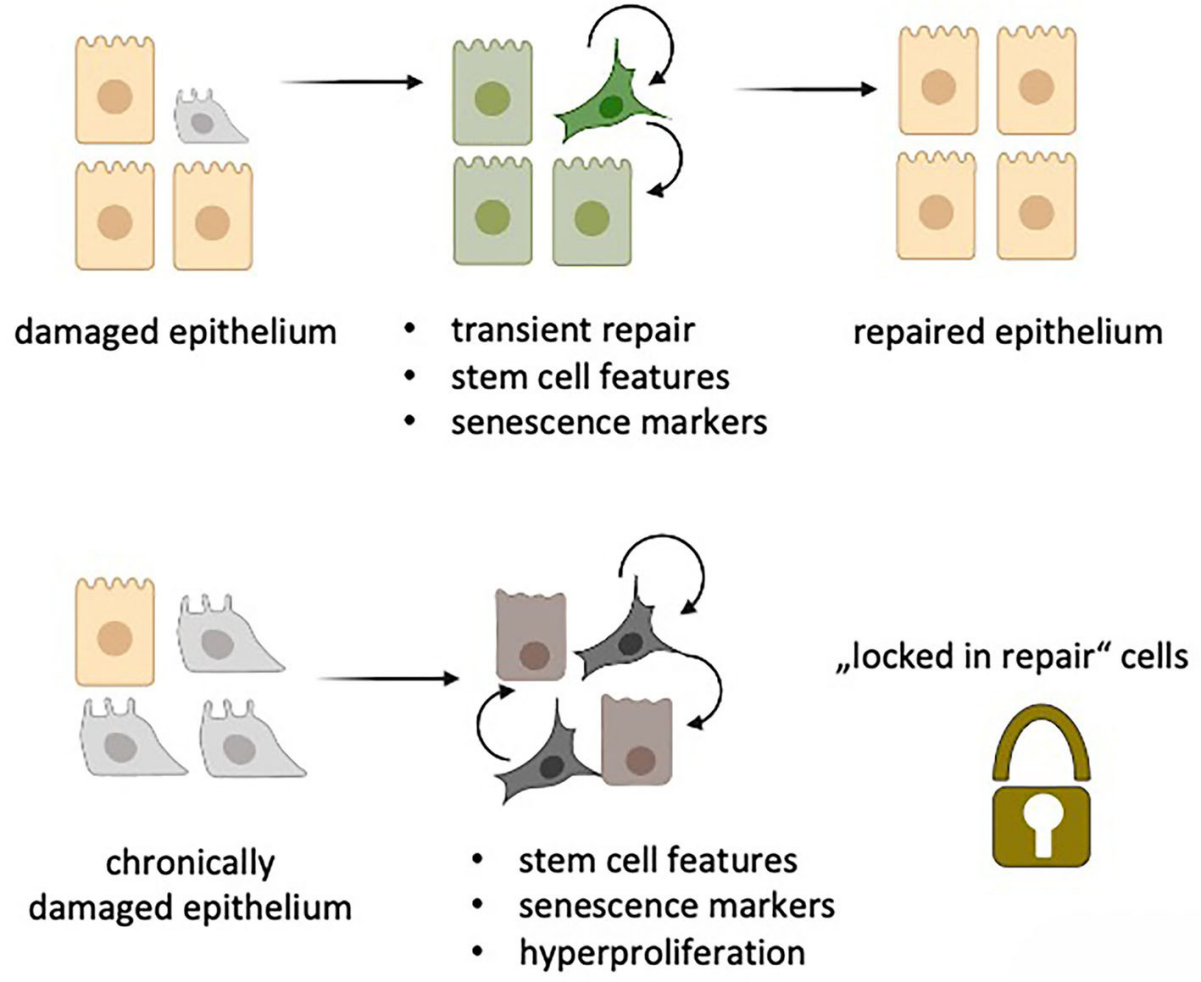
“Locked in repair” concept: Upon damage of the lung epithelium, transient repair cells (green) obtain features of senescence with stem cell like activities thereby facilitating repair of the damaged epithelium via autocrine and paracrine SASP secretion. Upon chronic epithelial damage, a similar process is induced. The senescent repair cells, however, are unable to repair the epithelium but rather promote hyperproliferation by maintaining stem cell like features via autocrine and paracrine SASP signaling as well as by escaping immune cell-mediated clearing. (Created in Biorender.com).

Given this astonishing overlap of gene expression programs between aberrant basaloid cells in IPF and the newly identified alveolar repair cells, i.e., de-differentiation, stemness and senescence, it is tempting to speculate that the IPF-specific basaloid cells are “locked in repair” (Figure 1). Locking of lung epithelial cells in a de-differentiated, plastic and senescent state would promote uncontrolled spreading of cellular plasticity and senescence to neighboring cells. Further, it would initiate maladaptive repair by continuous secretion of SASP-related inflammatory mediators and pro-fibrotic molecules. As senescent cells are inherently resistant to apoptosis they will not be cleared by cell intrinsic death programs (2). Moreover, senescent cells might also escape immune cell clearance by NK and CD8^+^ T cells (43). Together with defective immune surveillance in aging and chronically inflamed lungs (18) this will further increase the number of senescent cells thereby closing the vicious cycle that drives irreversible lung fibrosis in IPF. It might also contribute to an increased tumor burden observed in IPF patients (44).

## Discussion

Our concept predicts that repair-locked senescent cells represent an Achilles heel for the development of pulmonary fibrosis. Novel therapeutic concepts should then aim at targeting these cells in IPF by either eliminating them or putting them back on the right repair track. Such approaches could have both cell-autonomous as well as non-autonomous effects due to the expected modulation of the SASP.

The key issue is to understand what locks these cells in their senescent repair modus. Is this an irreversible state? Are there any means to unlock them? Comparison of these basaloid cells in IPF that seem to be permanently locked in repair with their repair competent counterpart in the bleomycin mouse model might help to shed light on possible approaches, including epigenetic signatures, to unlock these cells.

As the senescence program is characterized by extensive chromatin remodeling with predominant H3K9me^3^ histone signatures (2, 3, 15) it is well-feasible that distinct epigenetic changes mediate the repair-locked phenotype of aberrant basaloid cells. This hypothesis can be tested by investigating the chromatin landscape of basaloid cells and analyzing the effects of inhibitors of histone modifying enzymes on their phenotype. In addition, one should investigate the interaction with other cell types of the lung such as myofibroblasts and immune cells to distinguish cell-autonomous vs. non-autonomous effects. Moreover, the senescent program of aberrant basaloid cells in IPF might be specifically targeted by anti-senescent therapies such as senotherapeutics (45). Among these are senolytic compounds that aim to overcome apoptosis-resistance in senescent cells (46). Indeed, first in human trials demonstrated initial tolerability in IPF patients (47). However, these drugs do not discriminate reparative from aberrant senescence. A more specific approach might involve the development of engineered T-cells to selectively ablate senescent cells in a disease specific manner (48). This approach illustrates the heterogeneity of cellular states which is lumped together as “senescence” (5). For the development of a specific anti-senescent therapy in IPF, the lack of deep knowledge on the specific senescence phenotype including the SASP is the main hurdle that needs to be overcome. Moreover, any successful therapeutic approach would most probably be applicable to also familial forms of IPF and even other age-related lung diseases such as COPD (27). It's time to re-think targeting of senescent cells for therapy of chronic lung diseases.

## Author Contributions

All authors listed have made a substantial, direct and intellectual contribution to the work, and approved it for publication.

## Conflict of Interest

The authors declare that the research was conducted in the absence of any commercial or financial relationships that could be construed as a potential conflict of interest.
